# A qualitative study on the adoption of the new duty hour regulations among medical residents and faculty in Korea

**DOI:** 10.1371/journal.pone.0301502

**Published:** 2024-04-11

**Authors:** Eui-Ryoung Han, Eun-Kyung Chung

**Affiliations:** Department of Medical Education, Chonnam National University Medical School, Gwangju, South Korea; Kasturba Medical College Mangalore, Manipal Academy of Higher Education, INDIA

## Abstract

Duty hour regulations (DHRs) were enforced in 2017 in Korea to prevent the detrimental effects of excessively prolonged working hours among medical residents. We investigated the adoption of and implications of the new DHRs among medical residents and faculty members. Semi-structured interviews were conducted with 15 medical residents and 9 faculty members across general surgery, internal medicine, obstetrics–gynecology, and pediatrics departments at Chonnam National University Hospital. Based on the constructivist grounded theory, we developed themes from the data by concurrent coding and analysis with theoretical sampling until data saturation. In addition, respondent validation was used to ensure accuracy, and all authors remained reflexive throughout the study to improve validity. The methods of DHRs adoption among residents and faculty members included the following 4 themes: DHRs improved work schedule, residents have more time to learn on their own, clinical departments have come to distribute work, organization members have strived to improve patient safety. Residents have undertaken initial steps towards creating a balance between personal life and work. Teamwork and shift within the same team are the transitions that minimize discontinuity of patient care considering patient safety. Teaching hospitals, including faculty members, should ensure that residents’ work and education are balanced with appropriate clinical experience and competency-based training.

## Introduction

The duty hour regulations (DHRs) have been implemented to reduce resident burnout and to allow them to maintain a healthy work-life balance [1–3]. DHRs were introduced in Korea in 2017 and restricted the maximum work hours to 80 hours per week averaged over a 4-week period, the maximum hours of consecutive duty to up to 36 hours, and mandated a minimum off-duty time between shifts to 10 hours. Before the implementation of DHRs, medical residents in charge of critically ill inpatients were required to stay in the hospital for up to 24 hours a day, with no time off hospital calls [4]. Sleep deprivation from excessively prolonged working hours significantly increases the risk of medical errors by junior residents [5–7]. Therefore, improving the residency training environment by allowing for adequate sleep time for residents considering patient safety and quality resident education is crucial.

Although the DHRs were proposed to improve the residency training environment, residents and faculty members were concerned about poorer resident education and patient care quality [8, 9]. On the other hand, in contrast with the concerns, a study reported no significant differences in examination performance of the residents and patient clinical outcomes such as mortality or readmission rates before and after the implementation [10–12]. The systematic review of the literature has revealed the benefits of resident wellness after the 80-hour duty limit, but its effect on patient safety is inconclusive; furthermore, resident education in terms of objective and perceived impact has worsened or remained unchanged [13, 14]. Therefore, in countries implementing the DHRs, qualitative research has been conducted for a more comprehensive understanding of the perspectives and experiences of medical residents and faculty members, and the social contexts in which they are embedded [15–18]. In each country, a diversity of perspectives on the changes in residents’ lives and work, including fatigue and post-call behaviours, resident education, and patient safety, was studied. Depending on social context, different experiences and perspectives on the effects of DHRs may exist, but qualitative studies have not yet investigated them in our context [3, 19]. Because of an excessive patient load at teaching hospitals and a rigid organizational culture that forces residents to perform unnecessary tasks other than patient care, concerns have been raised as to whether residents’ working hours can be shortened even in our social context before the implementation of DHRs [19, 20].

With the implementation of DHRs, junior residents who earlier worked an average of ≥ 100 hours per week are now required to work <80 hours per week [4]. Hence, the junior residents will have more free time than before, and the senior residents will tend to work longer hours compared to the previous senior residents because they will be responsible for some of the tasks of junior residents. Teaching hospitals, including supervisors, should comply with the regulation to ensure that the residents’ rights are protected, resident education is not neglected, and patient safety is not compromised. Studies have reported a decline in residents’ working hours after DHR implementation, but in our context, the period of DHR implementation is still short; therefore, limited information is available regarding the experience of residents and the coping styles of residents and faculty in a new and completely different working environment [3, 19]. This study investigated how residents and faculty members at Chonnam National University Hospital, which is a regional-hub public medical institution, are adapting to DHRs and identified the challenges that remain to be solved.

## Methods

To explore the socially situated nature of these research questions, we adopted a qualitative design of the constructivist grounded theory in the form of individual semi-structured interviews by using a constant comparative method. We performed data collection and data analysis simultaneously, with informing the subsequent data collection. Using constant comparison methods, key concepts were continuously refined in an ongoing process of data coding until data saturation, meaning that no new insights emerged.

The study was approved by the Chonnam National University Hospital Institutional Review Board (IRB No. CNUH-2017-003).

### Participants

Medical residents and faculty members were recruited from Chonnam National University Hospital in April 2019. We targeted participants from major clinical departments including general surgery, internal medicine, obstetrics–gynecology, and paediatrics. They are in charge of critically ill patients and numerous inpatients, and therefore, they experienced the largest fluctuations in working hours before and after DHRs.

Since senior residents (who have experienced before and after DHRs) and junior residents (who are training after DHRs) may have different experiences and perspectives, we conducted interviews with residents from different training years in each clinical department. We sent emails containing an interview invitation to a total of 76 residents and encouraged participation through text messages. The resident interviews were conducted after confirmation of the participant’s willingness to participate.

After sending an official letter of cooperation to the aforementioned four clinical departments, the faculty interviews were conducted based on the recommendation of each department. The faculty members, recommended by respective departments, were in charge of the residency schedule and managing resident education in their clinical department. If additional explanation or clarification was needed, the next interview was conducted with a faculty member recommended by the previous interviewee. The total number of faculty members who were eligible for the interview was 89.

Informed written consent was obtained from all the interviewees. Finally, 15 residents (8 junior residents and 7 senior residents [in the third year or higher]) and 9 faculty members were included in the study.

### Data collection

A semi-structured interview guide was devised by two authors (S1 Appendix). Qualitative research is grounded to understand the diversity of experiences and perspectives [21]. An interview guide was constructed on the basis of previous studies in countries with different contexts but with work regulations and our previous findings implemented in the same social context [3, 8, 9, 15–18]. In-depth interviews focused on the effects of DHRs on the quality of the resident’s life, the resident’s education, and patient care, and how residents and faculty members coped with the change.

One of the authors conducted face-to-face interviews with each participant between May and June 2019. One interviewer author completed the residency training at the same university hospital and currently manages the clinical clerkship for medical students in respective clinical departments. The second author, independent of a residency program, is an expert in medical education and helped balance data interpretations during analysis.

Interviews ranged from 30 to 50 minutes with an average time of 43.2 minutes per sessions. Each interview was audio-recorded and transcribed verbatim. After each transcription was completed, the transcript was sent to the participant by email to ensure the correct meaning. For omitted or ambiguous expressions, the participant was asked to add a more detailed description. Among the participants, 5 out of 15 residents and 4 out of 9 faculty members further described the background and reasons behind some of the utterances. Thereafter, the anonymized transcripts were repeatedly analyzed and the interview scripts were revised for subsequent interviews.

Interviews were conducted using open-ended questions and clarification was sought from the participant if the interviewer could not fully understand or had any follow-up questions related to the interviewee’s answers. During the interviews, we simultaneously analyzed the collected data and made decisions about how to conduct the next interview. When a new topic emerged that required further exploration, we recruited additional participants who could reframe the question for clarification and provide contrasting perspectives.

### Data analysis

We analyzed the anonymized transcripts iteratively using a constant comparative method [21–23]. Each researcher read the interview transcript individually to identify key concepts in the texts and develop a code draft. We met to review the initial code set and reconcile any differences in coding. During the interviews, we read each new interview and compared the concepts conveyed in the new interviews with existing codes and categories. When there was no consensus or a new concept failed to meet an existing code or category, we returned to the original transcripts for review and reinterpretation. In the four data analyses where consensus could not be reached, we invited the relevant participants to a second session of member checks. We presented the analysis results via email and listened to their comments. Based on this exercise, the codebook and categories were iteratively revised and refined to include the themes. We continually sorted the collected data, analyzed and coded the information, and reinforced theory generation through theoretical sampling. We stopped collecting data when no new information could be obtained.

To minimise researcher bias, we used respondent validation, wherein participants checked their own interview transcripts. All researchers were constantly aware of their personal perspectives in research process and reflected on how these perspectives may have influenced the research strategies and results. Through open discussion, we reverified that the results align with the research aim to answer the research questions.

## Results

The interview results were classified into 4 themes on how residents and faculty members were adapting to the new DHRs. Table 1 showed an overview of the emerging categories and themes from the codes. In each section, the opinions of the representative participants for each theme were reported.

**Table 1:** Overview of the emerging categories and themes from the codes.

Codes (examples)	Categories	Themes
No calls (from the hospital)	Freedom from work	DHRs improved work schedule	
No worries (about being out)	Allowed free time		
Recovery while resting	Quality of life		
Focus on daytime work	Planned work and life		
Other schedules after work			
Discussion with faculty	Daytime benefit for learning	Residents have more time to learn on their own	
Operation, Procedures			
Preparation of next work	Utilizing allowed free time for learning		
Search for data			
Fellowship course	Extension of training time		
Task transferred to senior residents	Work redistribution to upward	Clinical departments have come to distribute work
Supervisor’s night shift		
Rotation of nightshift within a team	Effective team approach	
Cooperation (with other departments)	Hospital system in need of improvement	
Institutional support (of on-off system)		
Less tired	Fewer mistakes	Organization members have strived to improve patient safety	
Senior residents in charge of intensive care	Reasonable workload distribution within a team		
Not alone in nightshift (all training years residents on duty)			
Rotation of nightshift within a team			
Unchanged faculty role of final decision	Increased faculty role		
Increasing demands on faculty role			
Strengthen workforce of experts	Institutional support in need of reinforcement		
Increased awareness of patient safety			

The codes are presented with some representative examples. DHRs, Duty hour regulations

### 1. DHRs improved work schedule

The implementation of DHRs provided junior residents with regular off-duty time. Senior residents have experienced exhaustion due to critical patient care and unrelenting work when they were junior residents, but they expected that DHRs would provide junior residents with time for physical and emotional recuperation. Furthermore, the junior residents reported being able to organize their schedule after the end of day-work. Some junior residents reported being able to research on the patient’s condition and some others reported being able to better focus on preparing for a report or presentation on the next day.

The participants had the following responses about residents’ work schedule and lives after the DHRs.

“I have an allowed time, not to be blamed when I am out there.” (junior resident)

“Considering the situation in which I was never able to leave the hospital at the beginning of the first year, the current junior residents can put their cell phones down and rest for a while. Then, they could concentrate on their work the next day and afford to accept hospital calls.” (senior resident)

“The quality of life of residents now is much better than when I was a resident. Actually, it should have been like this.” (faculty member)

### 2. Residents have more time to learn on their own

As the allowed free time increased, the resident’s individual competencies depended more on self-directed learning. With the implementation of DHRs, the medical tasks concentrated on junior residents are evenly distributed among almost all residents, delaying the junior residents’ commitment to intensive care and tending to slow down the residency training process than before. DHRs seemed to allow residents to gradually adapt to their work. Faculty members opined that while basic core competencies can be acquired during the residency training course, a fellowship course will be required to acquire a higher level of knowledge and skills. Residents should improve their own knowledge and skills to avoid falling behind in the field after the residency training program. Some residents planned on undertaking a fellowship course to acquire advanced professional skills and gather more patient experience.

Regarding residency training because of reduced working hours, the participants had the following responses.

“Most patient care and treatment were done during the daytime, so I can discuss with my supervisor while I am awake and I can learn more.” (junior resident)

“Rather than seeing a lot of patients quantitatively, we should realize what we need to learn. In the end, I think it will depend on individual capabilities.” (senior resident)

“In comparison with a previous residency training course, the Korean Academy of Medical Sciences has developed the core competency of residents in detail and strengthened the evaluation, such as work-based assessment to determine their proficiency. I believe that active learning has become more possible and a requirement other than passive training because residents have more resources to acquire the medical knowledge they need, not just relying on patient experiences.” (faculty member).

### 3. Clinical departments have come to distribute work

The DHRs have facilitated changes in duty system and shift pattern. That is, by limiting the individual resident’s work hours to secure off-duty time and making patient care a team effort, work shifts progressed within a team including residents across course years. Few clinical departments are rapidly complying with the regulation. Within intensive care units or delivery rooms where physician presence is mandatory, the shift-based duty system has already been used. This duty system within a team was extended to other divisions within the same department after the DHRs implementation. To improve working conditions for residents and prevent dropout, this system was being applied to departments with a low annual resident recruitment rate, which had an insufficient number of residents. However, other departments in the transitional stage were of the view that coordination with the nursing department of the ward was necessary to communicate with the on-call resident after their work shift.

The participants expressed the following opinions regarding changes in work patterns caused by the reduction of individual resident working hours.

“Although the redistribution of work gave me more tasks than before, it was not difficult for me because of the faster decision-making and speed of processing.” (senior resident)

“I thought it was not unfair to work the night shift on behalf of the junior residents because of understanding the purpose of DHRs.” (senior resident)

“I would like the nursing department of the ward to check the next day’s prescription before the day shift resident leaves work in time for the takeover, and provide brief information about the patient when reporting to the night shift resident.” (junior resident)

“In order to prevent a gap in patient care, residents in charge and shift workers were assigned as a priority for the intensive care unit. So, there was no burden on a certain resident.” (faculty member)

### 4. Organization members have strived to improve patient safety

DHRs help eliminate immediate hazards to patient safety such as long working hours, although their long-term consequences on patient safety remain unclear. To redistribute the workload, junior residents were placed in charge of the general ward or the emergency room, and the senior residents were given charge of the intensive care unit. The night duty was rotated, with an even distribution of residents by training year (from the first to the fourth year). Patients in emergency were attended to by residents across all training years working at night rather than only by the junior resident in charge. Faculty members in the training hospital still made the final decisions on patient care and were more directly involved in patient care than before. Therefore, the quality of patient care during the training period remained unchanged.

As members of the organization, they presented the following opinions about the effect of DHRs on patient safety.

“It’s better now that a resident can sleep for a day and focus on his patient care the next day (senior resident)

“I am not alone in the ward late at night, so I can discuss the patient’s problems with my seniors.” (junior resident)

“The resident in charge was able to stay alert without getting tired because of the redistribution of workload. Although the residents’ work shift resulted in discontinuity of patient care, the negative impact caused by it was outweighed by the workload and intensity of their non-stop work on patient care.” (faculty member)

“I feel more reassured because senior residents are in charge of the intensive care units and they reside instead of on-call duty.” (faculty member)

“Considering the continuity of patient care, the working hours of faculty members should also be guaranteed by recruiting experts rather than distributing tasks within a limited workforce.” (faculty member)

## Discussion

The implementation of DHRs facilitated predictability of work hours for the residents and allowed residents to lead a planned life and provided them with time off of excessively prolonged work hours. Residents should achieve the goals set for each training course and actively learn the required skills. DHRs resulted in a transition to team-based patient care, with shifts including residents from each training year. DHRs resulted in teamwork between residents and faculty members, while eliminating potential hazards to patient safety. Despite the rigid hierarchical culture and excessive patient volume of tertiary hospitals, most residents complied with DHRs after their implementation [19]. The participants in this study agreed to the limits on work hours and were working within their organizations to ensure that appropriate residency training and patient care were not compromised.

The first theme is associated with the primary purpose of DHRs, which is improving residents’ work schedules. Improved quality of life for junior residents is the only theme achieving complete agreement [3, 8, 9]. Previously, residents used to take time off on their own and rest irregularly while on duty; however, after the implementation of DHRs, they could rest at a set time if they need a break and use their time as they planned. Junior residents could recuperate from physical and mental exhaustion because of excessively prolonged work hours. Before the limitation of work hours, many residents experienced sleep deprivation because of prolonged work hours. Severe sleep deprivation is associated with higher stress levels, impaired interpersonal relationships, and learning difficulties [24, 25]. Therefore, guaranteed rest is absolutely necessary to protect resident’s healthy life and education right.

The second theme is residents have more time to learn on their own. After the implementation of DHRs, residents work an average of 2 to 3 nightshifts per week; therefore, individual competencies will vary depending on how they spend their free time after regular work. Some residents seek relevant medical knowledge through clinical experience after work, and some residents plan for a fellowship course to acquire more advanced professional knowledge and skills after the residency program.

Although both faculty members and residents were concerned about the negative effects of work hour restrictions on resident education because of reduced clinical exposure, the majority of studies have found no measurable negative effects on the educational opportunities such as case volume for residents [26–28]. Furthermore, evidence supporting increased patient satisfaction and improved patient care quality because of a higher case volume or longer consultation is lacking [29–31]. In contrast, the residency training program requires providing for an appropriate balance between education and clinical experience without affecting sleep [26, 32]. That is, residency education should provide competency-based training that ensures the development of competent independent practitioners through self-directed learning rather than traditional fixed number of years in training [28, 33].

Thirdly, clinical departments have come to distribute work. A study reported a higher probability of a preventable adverse event when patients were treated by a physician not belonging to the primary care team [34]. To minimize discontinuity in patient care, work shifts occurred within a team that was well aware of the patient condition. Even before the implementation of DHRs, departments handling high severity or urgent cases were already using the duty rotation system within the team to ensure that the residents in charge had adequate sleep and could perform optimally. This system was being extended to departments with low resident recruitment rates to improve resident work conditions and prevent dropouts. Work shifts within the team minimized resident exhaustion compared to a traditional 24-hour call system, and improved the continuity in patient care [35, 36].

The last theme is organization members have strived to improve patient safety. The first step towards ensuring patient safety was the prevention of fatigue-related medical errors by preventing exhaustion among junior residents because of a heavy workload. The second step was a team-based approach to patient care with shifts within the same team. Faculty members played a central role in ensuring continuity in patient care and made the final decisions on patient care. The final step towards patient safety will be promoting the development of competent residents who continue to develop professional competencies along with appropriate patient experience [33, 37].

Our study has limitations. The sample was small and only included participants from a single institution. Therefore, we could not describe the differences in methods of adapting to DHRs by the hospital system and medical specialty. However, this study aimed to describe the attitudes towards accepting DHRs among those most affected by the change in working hours after the enforcement of the regulations. Another limitation is the focus on conformity given the study was conducted immediately after the enforcement of the regulations. Therefore, qualitative studies on addressing the advantages and concerns about working hour restrictions after DHRs are well established are required. Lastly, residents were encouraged to participate through individual text messages and faculty members through recommendations of the department and previous interviewee, however, the participation rate remained low. Data collection was terminated when reached data saturation and no new insights could be derived through continuous data analysis and coding.

## Conclusions

Medical residents have now taken their initial steps towards a balanced life and work. The working patterns of residents, including work shifts, have changed to teamwork in consideration of patient safety, and faculty members in teaching hospitals should ensure that residents have reached the minimum requirements of competencies for the year of training. That is, the education programs should be adjusted to ensure a balance between work and education combined with appropriate clinical experience and competency-based training.

## Supporting information

S1 Appendix A semi-structured interview guide. (DOCX)

S1 File (PDF)

## References

[1] Temple J. Resident duty hours around the globe: where are we now? BMC Med Educ. 2014;14(1):1–5. doi: 10.1186/1472-6920-14-S1-S8 25559277 PMC4304287

[2] Loo BKG, Ng CL, Chin RT, Davies LJ, Yong J, Ang AEL, et al. Nationwide survey comparing residents’ perceptions of overnight duty systems in Singapore: Night float versus full overnight call. Singapore Med J. 2020;61(10):559. doi: 10.11622/smedj.2020149 33225371 PMC7930313

[3] Han E-R, Chung E-K. The perception of medical residents and faculty members on resident duty hour regulation. Korean J Med Educ. 2020;32(1):67. doi: 10.3946/kjme.2020.154 32130852 PMC7066428

[4] Oh SH, Kim JS, Lee PS. A survey on training and working conditions of residents in 2015. J Korean Med Assoc. 2015;58(12):1179–89.

[5] Landrigan CP, Rothschild JM, Cronin JW, Kaushal R, Burdick E, Katz JT, et al. Effect of reducing interns’ work hours on serious medical errors in intensive care units. N Engl J Med. 2004;351(18):1838–48. doi: 10.1056/NEJMoa041406 15509817

[6] Nasca TJ, Day SH, Amis ES. The new recommendations on duty hours from the ACGME Task Force. N Engl J Med. 2010;363(2):e3 (1)–e3 (6). doi: 10.1056/NEJMsb1005800 20573917

[7] Lin Y-H, Ho Y-C, Lin S-H, Yeh Y-H, Liu C-Y, Kuo TB, et al. On-call duty effects on sleep-state physiological stability in male medical interns. PLoS One. 2013;8(6):e65072. doi: 10.1371/journal.pone.0065072 23750232 PMC3672167

[8] Drolet BC, Christopher DA, Fischer SA. Residents’ response to duty-hour regulations—a follow-up national survey. N Engl J Med. 2012;366(24):e35. doi: 10.1056/NEJMp1202848 22646511

[9] Drolet BC, Khokhar MT, Fischer SA. The 2011 duty-hour requirements—a survey of residency program directors. N Engl J Med. 2013;368(8):694–7. doi: 10.1056/NEJMp1214483 23425162

[10] Rajaram R, Chung JW, Jones AT, Cohen ME, Dahlke AR, Ko CY, et al. Association of the 2011 ACGME resident duty hour reform with general surgery patient outcomes and with resident examination performance. JAMA. 2014;312(22):2374–84. doi: 10.1001/jama.2014.15277 25490328

[11] Patel MS, Volpp KG, Small DS, Hill AS, Even-Shoshan O, Rosenbaum L, et al. Association of the 2011 ACGME resident duty hour reforms with mortality and readmissions among hospitalized Medicare patients. JAMA. 2014;312(22):2364–73. doi: 10.1001/jama.2014.15273 25490327 PMC5546100

[12] Rajaram R, Chung JW, Cohen ME, Dahlke AR, Yang AD, Meeks JJ, et al. Association of the 2011 ACGME resident duty hour reform with postoperative patient outcomes in surgical specialties. J Am Coll Surg. 2015;221(3):748–57. doi: 10.1016/j.jamcollsurg.2015.06.010 26228013

[13] Ahmed N, Devitt KS, Keshet I, Spicer J, Imrie K, Feldman L, et al. A systematic review of the effects of resident duty hour restrictions in surgery: impact on resident wellness, training, and patient outcomes. Ann Surg. 2014;259(6):1041. doi: 10.1097/SLA.0000000000000595 24662409 PMC4047317

[14] Philibert I, Nasca T, Brigham T, Shapiro J. Duty-hour limits and patient care and resident outcomes: can high-quality studies offer insight into complex relationships? Annu Rev Med. 2013;64:467–83. doi: 10.1146/annurev-med-120711-135717 23121182

[15] Taylor TS, Nisker J, Lingard L. To stay or not to stay? A grounded theory study of residents’ postcall behaviors and their rationalizations for those behaviors. Acad Med. 2013;88(10):1529–33. doi: 10.1097/ACM.0b013e3182a31157 23969351

[16] Taylor TS, Teunissen PW, Dornan T, Lingard L. Fatigue in residency education: understanding the influence of work hours regulations in Europe. Acad Med. 2017;92(12):1733–9. doi: 10.1097/ACM.0000000000001831 28746075

[17] Kreutzer L, Dahlke AR, Love R, Ban KA, Yang AD, Bilimoria KY, et al. Exploring qualitative perspectives on surgical resident training, well-being, and patient care. J Am Coll Surg. 2017;224(2):149–59. doi: 10.1016/j.jamcollsurg.2016.10.041 27884806

[18] Brown M, Tucker P, Rapport F, Hutchings H, Dahlgren A, Davies G, et al. The impact of shift patterns on junior doctors’ perceptions of fatigue, training, work/life balance and the role of social support. Qual Saf Health Care. 2010;19(6):e36. doi: 10.1136/qshc.2008.030734 21127102 PMC3002836

[19] Sohn S, Seo Y, Jeong Y, Lee S, Lee J, Lee KJ. Changes in the working conditions and learning environment of medical residents after the enactment of the Medical Resident Act in Korea in 2015: a national 4-year longitudinal study. J Educ Eval Health Prof. 2021;18. 33873263 10.3352/jeehp.2021.18.7 PMC8118751

[20] Chang H-J, Lee Y-M, Lee Y-H, Kwon H-J. Causes of resident lapses in professional conduct during the training: a qualitative study on the perspectives of residents. Med Teach. 2017;39(3):278–84. doi: 10.1080/0142159X.2017.1270432 28019136

[21] O’Brien BC, Harris IB, Beckman TJ, Reed DA, Cook DA. Standards for reporting qualitative research: a synthesis of recommendations. Acad Med. 2014;89(9):1245–51. doi: 10.1097/ACM.0000000000000388 24979285

[22] Kolb SM. Grounded theory and the constant comparative method: Valid research strategies for educators. J Emerg Trends Educ Res Policy Stud. 2012;3(1):83–6.

[23] Cristancho SM, Goldszmidt M, Lingard L, Watling C. Qualitative research essentials for medical education. Singapore Med J. 2018;59(12):622. doi: 10.11622/smedj.2018093 30009321 PMC6301871

[24] Kim HJ, Kim JH, Park K-D, Choi K-G, Lee HW. A survey of sleep deprivation patterns and their effects on cognitive functions of residents and interns in Korea. Sleep Med. 2011;12(4):390–6. doi: 10.1016/j.sleep.2010.09.010 21388879

[25] Baldwin DC Jr, Daugherty SR. Sleep deprivation and fatigue in residency training: results of a national survey of first-and second-year residents. Sleep. 2004;27(2):217–23. doi: 10.1093/sleep/27.2.217 15124713

[26] Peets A, Ayas NT. Restricting resident work hours: the good, the bad, and the ugly. Crit Care Med. 2012;40(3):960–6. doi: 10.1097/CCM.0b013e3182413bc5 22297631

[27] Levine AC, Adusumilli J, Landrigan CP. Effects of reducing or eliminating resident work shifts over 16 hours: a systematic review. Sleep. 2010;33(8):1043–53. doi: 10.1093/sleep/33.8.1043 20815185 PMC2910534

[28] Moonesinghe S, Lowery J, Shahi N, Millen A, Beard J. Impact of reduction in working hours for doctors in training on postgraduate medical education and patients’ outcomes: systematic review. BMJ. 2011;342. doi: 10.1136/bmj.d1580 21427046

[29] Elmore N, Burt J, Abel G, Maratos FA, Montague J, Campbell J, et al. Investigating the relationship between consultation length and patient experience: a cross-sectional study in primary care. Br J Gen Pract. 2016;66(653):e896–e903. doi: 10.3399/bjgp16X687733 27777231 PMC5198642

[30] Wilson AD, Childs S. Effects of interventions aimed at changing the length of primary care physicians’ consultation. Cochrane Database Syst Rev. 2006;(1). doi: 10.1002/14651858.CD003540.pub2 16437458

[31] Choudhry NK, Fletcher RH, Soumerai SB. Systematic review: the relationship between clinical experience and quality of health care. Ann Intern Med. 2005;142(4):260–73. doi: 10.7326/0003-4819-142-4-200502150-00008 15710959

[32] Blum AB, Shea S, Czeisler CA, Landrigan CP, Leape L. Implementing the 2009 Institute of Medicine recommendations on resident physician work hours, supervision, and safety. Nat Sci Sleep. 2011;3:47. doi: 10.2147/NSS.S19649 23616719 PMC3630963

[33] Long DM. Competency-based residency training: the next advance in graduate medical education. Acad Med. 2000;75(12):1178–83. doi: 10.1097/00001888-200012000-00009 11112714

[34] Petersen LA, Brennan TA, O’Neil AC, Cook EF, Lee TH. Does housestaff discontinuity of care increase the risk for preventable adverse events? Ann Intern Med. 1994;121(11):866–72. doi: 10.7326/0003-4819-121-11-199412010-00008 7978700

[35] Shirreff L, Shapiro JL, Yudin MH. Perceptions of a night float system of resident call within an obstetrics and gynaecology residency training program. J Obstet Gynaecol Can. 2014;36(11):957–61. doi: 10.1016/S1701-2163(15)30407-2 25574671

[36] Yu HW, Choi JY, Park YS, Park HS, Choi Y, Ahn S-H, et al. Implementation of a resident night float system in a surgery department in Korea for 6 months: electronic medical record-based big data analysis and medical staff survey. Ann Surg Treat Res. 2019;96(5):209–15. doi: 10.4174/astr.2019.96.5.209 31073510 PMC6483933

[37] Ten Cate O. Trust, competence, and the supervisor’s role in postgraduate training. BMJ. 2006;333(7571):748–51. doi: 10.1136/bmj.38938.407569.94 17023469 PMC1592396

