# Supplementary material for: A qualitative study on the adoption of the new duty hour regulations among medical residents and faculty in Korea
Source: PLoS One. 2024 Apr 11;19(4):e0301502. doi: 10.1371/journal.pone.0301502 (PMC11008864; doi:10.1371/journal.pone.0301502)
Supplement: S1 Appendix — (DOCX) [file pone.0301502.s001.docx]

S1 APPENDIX: A Semi-Structured Interview Guide

Resident duty hour regulations (DHRs) have been implemented since 2017 with three purposes: to protect residents’ rights, contribute to high-quality resident education, and improve patient safety. An in-depth interview will focus on the effects of DHRs on the residents’ quality of life, education, and patient care. I will ask about your thoughts on these effects and the coping styles you adopted with such changes.

1) Residents’ life

- (junior residents) How about your work schedule?

- (senior residents) What are the changes in your work life after DHRs?

- (faculty members) What do you think about the residents’ quality of life after DHRs?

- (Prompt) Your division has been running an off-duty time even before DHRs. Has anything changed after that?

- (Prompt) More on-call duty or inpatient care was added after DHRs. What do you think?

2) Resident education

- (Residents) How about your patient experience?

- (All) What do you think of residency training after DHRs?

- (Prompt) There are concerns that education is not enough like before (insufficient training time or lack of patient experience, etc.). What do you think?

- (Prompt) How do you enhance your competence?

3) Patient safety

- (All) What do you think is the impact of DHRs on patient safety?

- (Prompt) How do you think the changed residents’ work schedule (you mentioned) will affect resident education?

- (Prompt) How do you think the aforementioned resident competencies will affect patient care?
