# Supplementary material for: A qualitative study on the adoption of the new duty hour regulations among medical residents and faculty in Korea
Source: PLoS One. 2024 Apr 11;19(4):e0301502. doi: 10.1371/journal.pone.0301502 (PMC11008864; doi:10.1371/journal.pone.0301502)
Supplement: S1 File — (PDF) [file pone.0301502.s002.pdf]

Interview transcripts (Q & A)<br>(translated from Korean to English)	Code drafts
Q: How has your work schedule changed after the introduction of duty hour regulations (DHRs)?<br>A: It is not easy to compare with previously trained residents; however, when I hear of their past work lives, I definitely get more sleep and am not permanently at the hospital.	Improved work–life balance.
Q: How has work been since the DHRs were introduced?<br>A: Working hours are from 8:00 am to 8:00 pm, six days a week (Sunday to Friday, and only four hours on Saturdays). During emergency room duties, I report ER patients to the next resident on duty and hand over the phone number of the next resident to patients to ensure all calls go directly to him. Patients seen during emergency room duty hours but not yet admitted are also transferred to the next resident while they remain in the emergency room.	No calls, complete freedom from work.
Q: How many hours per week do you work following the introduction of the DHRs?<br>A: We adhere to a strict 80-hour workweek limit. Even during night shifts, we ensure that consecutive work hours are less than 36, with a guaranteed 10-hour rest period between shifts. When covering emergency room shifts on weekdays, we work 60–70 hours per week because we work night shifts every three days, with the remaining two days spent on daytime shifts, resulting in less than 70 working hours per week. Even first-year residents end up working a total of 78 hours. Often, the remaining time is spent on self-study; if there are pending tasks after work, some residents may stay until 10 pm to complete them, during which time they could receive calls to cover the ward. After finishing these tasks, we formally request for the next attending physician to handle any further responsibilities. However, for patient safety and as a sense of responsibility as the attending physician, residents may stay back to attend to specific cases, such as adjusting insulin for diabetic patients. While first-year residents are usually assigned to inpatient care following staffing shortages, third-year residents sometimes take on this role once every six days, with third-year residents also overseeing ICU care. Interestingly, rather than covering both ER and ICU, each area has designated attendings, and residents from different departments take turns covering shifts. In the past, second-year residents would sometimes assist with ER and ICU duties. Subsequently, if multiple patients were critical across departments, assistance would be requested from attending physicians in various specialties. After completing a daytime shift and staying up all night due to critical patient cases, residents would often feel physically exhausted and experience burnout, while others would resume their normal routines the following day.	Allowed free time.<br>Any schedule after work is possible.<br>Patient safety is a priority.<br>Moral responsibility.<br>The task was transferred to senior residents.<br>Senior residents are in charge of the ICU.<br>Rotation of night shift within a team.<br>Physical recovery.
Q: What do you think about the changes at work after the DHRs were introduced?<br>A: As a current third-year resident, with the third-year workload increasing and even	Agree to improve residents' lives.

after enduring the challenges of being a first-year resident, my honest personal sentiment is that I would not say I like having to repeat the same tasks all over again. However, I understand the intention behind this regulation, as I felt how difficult it was to handle all of this alone during my first year. Moreover, since I am not handling as much of the burden as I used to, I feel that sharing the workload to some extent is more reasonable, and I accept that establishing this system is more valid.	
Q: In the XXX department, is there a practice of working for 36 consecutive hours, considering that there are clear ON/OFF shifts with 10-hour shifts (from 8 am to 6 pm) and 14-hour off periods? In other departments, it has been known for workers to transition directly from a 10-hour shift to an on-call duty. How does it work in the XXX?<br>A: Until last year, XXX had a similar setup, but this year it has been changed so that there is a day off after the on-call duty. (Then who takes over the responsibilities during this time?) Previously, the chief resident had little else to do during the daytime besides entering the operating room. However, since surgeries are not conducted every day in the XXX, the chief ends up having more tasks to handle. The attending physician takes over the call duty when the chief is off. This means the third and fourth-year residents end up taking on more responsibilities. Initially, in the XXX, both the attending physician and the chief would take turns serving on-call duty to ensure that serving the on-call duty while handling ward calls does not become a significant burden. The role of the chief is to supervise under the guidance of the professor and to rotate through the wards to see patients along with the attending physician. Of course, some chiefs did not fulfill this role properly, but attending to patients in the ward was traditionally considered part of the chief's duties.	The task was transferred to senior residents.
Q: How has your work changed after the DHRs were introduced?<br>A: Currently, as a third-year resident, I also take on the role of an attending physician. Compared to before, there has been an increase in the number of third-year attending physicians. Previously, it used to be XXX, XXX, etc., although it has now gradually expanded. Residents from the first to fourth years are paired up to work together. Sometimes, fourth-year residents even handle around 20 assigned patients. In the XXX division, fourth-year residents may see more patients, while in the XXX division, the workload is sometimes divided in half.	The task was transferred to senior residents.<br>Duty system within a team.
Q: How is life as a senior resident after the implementation of the DHRs?<br>A: Well, as I have progressed through the years, despite the increased workload, my experience and knowledge have made the tasks quicker and decisions easier, meaning they are more manageable. Interestingly, while it used to be quite challenging for first-year residents to handle the neonatal unit alone, instead of working six days a week with some extra duty, second- and third-year residents each take turns with night shifts,	The task was transferred to senior residents.<br>Rotation of night shift within a team.<br>Increased awareness

which is definitely better for the well-being of the infants.	of patient safety.
Q: How has life changed for residents after the DHRs?<br>A: While third-year residents work night shifts more often than before, schedules are structured to ensure we stay within 80 hours per week. This allows for breaks during emergency room shifts, ensuring we do not exceed 36 hours on duty and have a day off. Though there are additional responsibilities as we progress in seniority and specific tasks within each department, it is not overwhelmingly exhausting to the point of feeling like we might die. Getting proper rest enables us to focus better and perform our duties more effectively the next day, which is an improvement.	The task was transferred to senior residents.<br>Breaks.<br>No unfair work.<br>Focus on daytime work.
Q: How has work been since the implementation of the DHRs?<br>A: We work from 8 am to 6 pm, starting at 7:30 and usually finishing around 7 to 8 pm. After work, I am not responsible for inpatient care, so I do not receive calls from the hospital during my free time. Notably, the workload has increased compared to before, when third-year residents only worked in outpatient clinics (without on-call duties). However, when I think about how, during my first year, I could not leave the hospital for 3–4 months straight while working on the wards, it is crucial to take breaks. Remembering how, even during my second year, I could barely rest after shifts in the ER/ICU, I realize that life in the first and second years has improved significantly owing to the assistance from the third and fourth years. While it is frustrating having to redo tasks, ultimately, it is not unfair.	The task was transferred to senior residents.<br>Breaks.<br>No unfair repetitive work.
Q: How has life been for residents after the DHRs?<br>A: Prior to the 80-hour working limit, there was a unique specialty area called XXX, where attending physicians had to be present all day, unlike in other departments. Therefore, even before the 80-hour limit, we were rotating shifts similarly. Life as a resident has mostly stayed the same after the introduction of the 80-hour limit. During off-hours, no calls are received. For the division of XXX, the ON/OFF system was clear even before the 80-hour limit. There is a duty room within the ward, where we are on standby instead of caring for patients on call. (Since when?) From about 10 years ago? Perhaps. If the handover is smooth, we strictly adhere to the 7 am to 6 pm working hours. During the daytime shift, all residents from years 1 to 4 would be on duty, with first-years on standby, reporting to second-years, and if issues were not resolved, then to the chief. (Does the first-year resident work both day shifts and on-call shifts?) On the days they are on duty, they work from the daytime to overnight. They work for 24 hours and have 24 hours off on weekends. Initially, first-year residents would leave on Friday evening and return on Sunday morning. However, due to the 80-hour limit (and to avoid one person working continuously for 48 hours), they now have Saturdays or Sundays off and take 2–3 weekdays off. Eventually, first-year residents are working within the 80-hour limit. (Do they work for two days and then	Rotation of night shift within a team.<br>No calls, complete freedom from work.<br>Physical recovery.<br>Preparation of next work.<br>The task was transferred to senior residents.<br>No unfair repetitive work.<br>Supervisor's night shift.

have a day off?) They have one day off and only have the daytime off during weekends. There has been little change for first-year residents. There have been changes for third-year residents, although they seem reasonable. Third-year residents can have days off, but serving on-call duty once every 2–3 days seems fair. They find it acceptable to serve on-call duty occasionally rather than having an entire day off, as it is not unfairly demanding. There has not been a significant change in the life of XXX residents, but they are now given definite off-hours. It seems the lives of attending physicians are definitely improving. They can get enough sleep and prepare for the next day. With the opening of XXX, the proximity of the wards has improved significantly. When XXX was located on X floor, and the XXX department was on X floor, with the move, they are now adjacent, allowing for immediate response when needed. XXX functions as a medical emergency (ME) ICU. To improve the lives of residents due to a decrease in applicants for the XXX department (due to unfilled quotas), they had their limits on working hours and implemented the ON/OFF system.	
Q: How do you think the lives of residents have changed since the DHRs?<br>A: Nowadays, with the training time limited to 80 hours per week, residents typically work for 12 hours on weekdays (from 8 am to 8 pm). They are ensured off-time after 8 pm and do not take calls. Continuous shifts are also kept under 36 hours. In other words, when they are on duty, they take a 10-hour break instead of waiting for calls then return. For emergency room shifts, they work for 12 hours and then must rest for the next 12 hours. They strictly adhere to the 80-hour limit in the XXX department as well. In my personal opinion, the quality of life for residents has improved significantly. Dealing with difficult patients usually meant staying up for 2–3 days straight because the patient's condition did not improve. However, since there is currently a break (resting period), residents experience less stress in dealing with patients. The stress they receive from their superiors (such as reprimands) is also reduced. Overall, it is much better.	Improved life.<br>Emotional recovery.<br>Breaks.
Q: Do you feel like the lives of residents have improved with the 80-hour working limit?<br>A: Definitely. The residents look less worn out and more cheerful. For new first-year residents, it took some time to adapt from March to April, but by May, after completing one rotation, they became more accustomed to the work, meaning the fatigue seems to have decreased. (How many hours do residents work?) They work from 8 am to 7 pm during the day shift, with second-year residents responsible for ward rounds and third-year residents managing the intensive care unit (ICU) from 8 am to 6 pm. Second or third-year residents also oversee the ICU. Now, there are no emergency room calls for ward residents. With separate emergency room attendings, second or third-year residents take charge. Operations are split by department and ward, meaning the shifts	Improved life.<br>Moral responsibility.<br>Any schedule after work is possible.<br>The task was transferred to senior residents.<br>Senior residents in charge of the ICU.

are determined by ward or department. Currently, they work ON/OFF shifts, working until night on duty days. After a 24-hour shift, they rest for 10 hours and then return. Continuous shifts last for 35 hours. After a 24-hour shift, they work a 12-hour day shift, accumulating 35 hours. Then, they rest for 13 hours and return. Duties are assigned according to the ward. They do two weekdays and one weekend duty per week in their first and second-year residencies. Third-year residents also take shifts. They are assigned and perform ward duties three times a week. The schedule is arranged so that third-year residents and backup fourth-year residents handle duties instead of attending the wards. There are also shared wards where second-year residents are in charge. Currently, the XXX department limits work hours to within 88 hours. Some wards stay within 80 hours, while others slightly exceed it. (Are residents in the XXX department leaving work exactly on time?) They seem to be staying about 1–2 hours overtime. It appears they are finishing up unfinished tasks before leaving. They seem to spend a little more time with inpatients to ease the burden for on-call residents. They seem to extend handover times to accommodate backup duties, but residents who need to leave for their OFF time do not currently seem to be wrapping up quickly. Fourth-years leave on time, while first and second-years stay until the handover is complete.	
Q: How do you feel about the life of residents after the DHRs?<br>A: The atmosphere among residents has brightened, and their expressions have improved. (Do you think they engage in self-learning during their free time?) It seems they do engage in self-learning. In the future, while receiving residency training, they feel they should set goals to learn something and make efforts on their own. They believe that if they do not actively seek learning opportunities, they will not learn anything and will stagnate. From the perspective of supervising specialists, it is overwhelming to educate each resident due to the increased workload. While they provide autonomy, residents must engage in self-directed learning, leading to differences in individual capabilities.	Improved life.<br>Self-directed learning is needed.<br>Depends on the capabilities of the individual.
Q: How do you find the life of residents after the Residency Law?<br>A: Much better. Compared to ten years ago, first-year residents had no days off until August. (Does that mean they only had off time during vacations?) There were no official days off until August. After taking a summer vacation in July–August, they could have days off from September, but there were no weekends off, only weekdays off. That started to change and improve gradually. With the implementation of the DHRs, there are now restrictions on working hours and duty shifts, allowing for more weekdays and even a day or more off on weekends. Additionally, the finishing time became clearly defined following the decrease in duty shifts. Additionally, being called in for on-call duty decreased and has now disappeared. In the past, even after leaving work, there were occasions when they were called back, but now that is gone, giving	Improved life.<br>No calls, complete freedom from work.

the residents more freedom once they leave the hospital. With clearer commuting times and no calls after leaving work, the quality of life has significantly improved.	
Q: How has life as a resident changed since the DHRs?<br>A: Compared to my residency seven to eight years ago, it is much better now. The ON/OFF system is clear, so there is no waiting around or sleepless nights, and the residents get free time. They start work at 8:30 am, coinciding with the morning conference, and leave at 5:00 pm, regardless of elective outpatient appointments. After that, surgical responsibilities are handed over to the on-call doctor. Considering the available workforce, first-year residents in the operating room either hand over to senior residents or colleagues in the same year. In the wards, one ward-duty doctor and one on-call doctor cover the emergency and operating rooms, meaning first- and third-year residents work together, whereas second- and fourth-year residents team up. (Is strict quitting time observed?) It is only sometimes strictly followed in some departments. Therefore, there was a decision within the XXX department to submit schedules adjusted according to specialties to comply with the 80-hour rule for residents. XXX division schedules are not well adhered to because of the heavy workload. Other divisions have only two clinical professors, but XXX has three. Due to the longer duration of XXX surgeries, there are areas where schedules are not well kept. We differentiate between weeks with XXX surgeries and weeks without them when organizing schedules. After our working hours, we immediately switch to shift work.	Improved life.<br>Duty system within a team.
Q: How has life changed since the DHRs?<br>A: One of the toughest parts during my first year was that previously, as the attending physician, I had to handle the ward alone (without any colleagues or shift workers), which was extremely challenging. Especially in the wards with many critically ill patients who could deteriorate at any moment, receiving calls in the middle of the night or early morning could lead to burnout. One positive aspect of the on-call system introduced by the DHRs is that after taking a day off, there is a sense of relief knowing that even if a call comes in the early hours, I have the flexibility to go and attend to it. Taking a break allows me to recharge and motivates me to handle tasks. (It is better for the attending physician and the patients.) If patient handover is performed effectively, I do not think patient safety would be compromised.	Rotation of night shift within a team.<br>Emotional recovery.<br>Residents' well-being is related to patient safety.
Q: How has life changed since the DHRs?<br>A: Life as a resident has definitely improved. I had no regrets about not having much personal time while serving as a resident in the first couple of years, as I anticipated it would be challenging to have my own time while on call. However, looking back now, as a fourth-year resident, I wish I had more leisure time or opportunities to study, which I have now. I cannot help but wonder how it would have been if I had received these	Other schedules after work are possible.<br>Concurrent self-directed learning is needed.

opportunities, even during my first year. It would have been nice to have guaranteed study time alongside work.	
Q: Has the working hours decreased for residents, but how about as a supervisor?<br>A: The workload for junior staff and fellows has increased significantly. When considering the workload for the future, it does not feel pleasant for those in their third or fourth year. Departments tend to recruit more fellows to meet the growing demands, which means more work. Personally, my working hours have decreased. However, the number of fellows has increased to match the residency quota, resulting in fewer on-call duties for individuals. As a supervising faculty, I am on call. I work about 2–3 times a week, so I cannot be away from the hospital too often.	Increased demands on the faculty roles.<br>Strengthen the workforce of experts.
Q: How has the workload for the supervising faculty been since the implementation of the DHRs?<br>A: Originally, we were on call regularly. Compared to before, we receive more phone reports now. I would say the quality of life for the supervising faculty has decreased. The workload for fellows has increased, and we anticipate that the workload for clinical professors and junior staff will also increase soon.	Increased demands on the faculty roles.
Q: With the limitation on working hours, has the workload decreased accordingly?<br>A: Since I was expected to be present in the hospital consistently, I used to work a little more loosely before the DHRs; however, presently, I need to finish within the allotted time, so I try to do things as quickly as possible, resulting in a similar overall workload as before. The quantity remains similar, but the pace has accelerated. Tasks that were previously postponed now seem to be resolved more promptly. However, due to hospital accreditation requirements, a considerable amount of time is spent on documentation and verifying numerous details, which calls for system improvements.	Unchanged workload.<br>Faster work speeds.<br>Institutional support is needed.
Q: Do you think the workload has decreased due to the 80-hour limit per week?<br>A: I do not think the workload has decreased. As long as the number of beds does not decrease, and there are no actual empty beds, there will continue to be similar workloads. In fact, the workload feels much heavier than before. Working through the EMR system has become more demanding with the additional inputs required. For instance, there are now many additional fields to fill out, such as pre- and post-procedure documentation and entries required to enhance clinical indicators. This results in more pop-up windows for confirmation after entering prescriptions. Third-year residents feel that although they are more proficient in doing the same tasks, there seems to be an increase in workload outside of patient care compared to before. While the number of assigned patients has definitely decreased (as distributed by third- and fourth-year residents), the additional administrative tasks have increased, meaning the total workload has not decreased significantly, if at all. We used to handle 40–50 patients in the past, but now it is 20–30. However, the amount of documentation	Unchanged workload.<br>Institutional support is needed.

generated by these patients has increased due to the expanded required fields. (What improvements do you think are needed?) First-year residents naturally need time to become familiar with the work, and while there are fewer patients than before, the ancillary tasks required as an inpatient provider have increased. Currently, it seems that streamlining and expediting processes in the EMR system are necessary.	
Q: When asked if the workload has decreased as the working hours have reduced, the residents mentioned that the workload has not decreased. They specifically mentioned that the time required for patient documentation has increased. What are your thoughts on this?<br>A: I believe it is inevitable. With the allocation of patients, there is a need to leave clear handover notes and details (both for continuity of care and legal protection). Additionally, with the EMR system, there are more confirmation prompts for each prescription, so it is more likely that the workload has increased rather than decreased. Although the number of patients they observe has decreased, I anticipate the documentation workload for patient care will increase further.	Unchanged workload.
Q: Is the ON/OFF system being implemented effectively?<br>A: Those who are off-duty do not receive any calls. Even off-duty residents do not answer calls from the wards, while the wards also do not call them. However, the time for leaving off-duty is still unclear, and many leave around 11 pm, especially when the resident has not finished organizing or when ward rounds have run late. Nevertheless, once they are off-duty, they do not receive any calls. (Do you know if residents who are not attending physicians also handle prescriptions for admitted patients the next day, and when do the ward nurses check this?) In the XXX department, the attending physician handles the prescriptions for the next day and then leaves. Thus, it seems appropriate for the prescription confirmation to be directed to the attending physician rather than the on-call resident. From the perspective of the resident on duty, they would take responsibility if something happened to the patient overnight, but it seems difficult for them to confirm prescriptions regularly. It would be helpful if the ward could confirm prescriptions more promptly.	No calls, complete freedom from work.<br>Any schedule after work is possible.<br>Cooperation with the nursing department.
Q: Is leaving work on time well-managed?<br>A: Even if I leave, I do not receive criticism or reprimand. There is now a time when I do not have to worry about leaving as soon as work is completed, allowing me to pursue my personal life. I believe previous shortcomings, such as being caught in emergency situations, have been improved.	No worries, complete freedom from work.
Q: I expected to be able to compare the training process of the second year with that of the first year, but I heard that even before the regulation imposing an 80-hour training limit for residents was enacted, XXX department had already been implementing an ON/OFF system, and there was no change in the ON/OFF system	No calls, complete freedom from work.

regardless of the enactment of the DHRs. Has anything changed in the ON/OFF system since the enactment of the DHRs?<br>A: XXX was implementing it even before the regulation was enacted. Before the regulation, during the first year, I received calls from the attending physician until midnight (12:00 am), but with the 80-hour limit, even the calls from the wards were no longer received. The ward duty takes all the calls, and the attending physician receives no calls after completing their working hours.	
Q: What are the working hours after the implementation of the DHRs?<br>A: Typically, we start at 8:00 am (conference) and work until surgery finishes around 6:00 pm. Actually, there was a difference between XXX and XXX divisions. In the XXX, calls from the attending physician used to come until midnight (12:00 am) before the regulation. However, in the XXX, even before the enactment of the regulation, calls were not received after working hours. There has not been any change in the XXX regarding the DHRs, but the XXX has changed now as well.	Expanded ON/OFF system.<br>No calls.
Q: Do you usually leave work right after your scheduled hours, or do you tend to stay longer at the hospital depending on personal tasks or the condition of patients in the ward?<br>A: I typically leave work once my scheduled hours are over. My workload is not usually so overwhelming that I need to stay beyond that. The on-call physician can handle any necessary prescriptions or care for patients in the ward during their shift, so I do not need to linger. XXX department currently has attending physicians rather than on-call residents taking emergency room calls. In other words, attendings are responsible for both the ward and the emergency room until the end of their shifts.	Guaranteed OFF time.<br>No calls, complete freedom from work.
Q: When you are off duty and leaving the hospital, do you not feel pressured when your assigned patients are not doing well or handovers are improperly organized?<br>A: I think it varies from person to person. Some may feel uncomfortable leaving without a proper handover and feel responsible for staying until things are sorted out. Others might leave promptly when their shift ends. Actually, there is usually no issue, but individuals feel a lingering sense of moral obligation or duty. From what I have heard in our department, even during emergency surgeries, when it is time for handover, the surgery continues, yet the following on-call physicians step in to assist. This practice seems to be well established. Initially, it might not have been the case. In the XXX department as well, attending physicians used to handle multiple areas alone, such as the ward, emergency room, and emergency surgeries. However, situations arose where they had to leave during emergency surgeries if there was a new emergency room patient, so protocols were established to address such situations. We often discuss how well the XXX department's on-call system is structured.	Moral responsibility.<br>No calls, complete freedom from work.<br>Institutional support of the on–off system is needed.
Q: How is the on-call system after the implementation of the DHRs?	Transition period.

A: It can vary depending on the department's characteristics. In departments with longer attending physician terms, strictly adhering to the on-call system as mandated by the regulation is often challenging. In the XXX department, where we have a high number of inpatients, it is not always feasible to hand over patients to the on-call physicians, similar to nurses with their duties. Consequently, patient handovers are not always meticulous. There are instances in the wards or intensive care units where they inevitably need to reach out to the attending physician again. While the resident's life has certainly improved, it does not currently represent a complete off-duty situation. Calls still come in during the early hours, and XXX is still in its early stages.	
Q: Was there any aspect of the ON/OFF system that helped establish it in departments like yours that had been implementing it even before the regulation was enacted?<br>A: Given the shortage of residents, faculty took the initiative to address the issue. The non-clinical department used to allow residents to take weekends off and even leave work early on regular days. However, our department, having faced the crisis of a shortage of residents, had already established a duty ON/OFF system, including improvements in training environments and faculty participation in duty, so we are currently proceeding smoothly without any difficulties.	Supervisor's night shift.
Q: How is the ON/OFF system after implementing the DHRs?<br>A: Last year, fellows voluntarily took on-call duties to adhere to the 80-hour weekly limit, thereby enabling us to proceed. Currently, with all positions in our department filled, schedules are organized to comply with the 80-hour regulation. However, in case of unforeseen circumstances, there is now a shift in the mindset of those senior supervisors. They are now willing to step in and cover shortages if they arise, showing consideration and a willingness to help.	Supervisor's night shift.
Q: Since when has this ON/OFF system been in place in the XXX department?<br>A: It has been implemented since the start of the DHRs. We began increasing the number of weekdays off and significantly expanded off-duty periods around 7–8 years ago. Initially, it was minimal but gradually increased. There were discussions about improving training conditions for the residents, so adjustments were made gradually, resulting in more off-duty time. The enforcement of the DHRs ensured a strict adherence to the ON/OFF system. Currently, third- and fourth-year residents work fewer hours than first- and second-year residents who work 80 hours per week. We could align the working hours by reducing the number of on-call physicians from four to three. (What does having three on-call physicians mean?) This means we distribute the on-call duties evenly among residents from the first to fourth year. We have been divided into parts, XXX, XXX, and XXX, and each part operates as a team. (Regarding concerns before the enactment of the DHRs, questioning whether handing over patients to on-call physicians, especially when patients are at critical points, would jeopardize	The task was transferred to senior residents.<br>Rotation of night shift within a team.<br>Increased awareness of patient safety.

patient safety, how does XXX department handle this?) Previously, there were fewer off-duty slots for attending physicians in the XXX department. Leaving would create gaps, and handing over patients was often challenging. However, to comply with the 80-hour regulation, attending physicians were on call about three times. With the team system in place, even if a first-year resident is on call, the second-year resident in the same part takes over, ensuring patient continuity, which has not posed significant issues.	
Q: Have there been any improvements in the ON/OFF system since the enactment of the DHRs?<br>A: We have implemented sending out on-call duty notification messages according to hospital shift schedules. Upon receiving the message, when sending a patient to the ward, we first contact the next on-call physician member (confirming the on-call physician first). There has also been internal gradual compliance with this system. Although we started without much preparation, we are now aligning with this system (viewing it ultimately as the right approach).	Institutional support of the on–off system is needed.
Q: Have there been any improvements in the ON/OFF system since the enactment of the DHRs?<br>A: Currently, a challenge is that ward nurses may not be aware of the resident's 80-hour limit, and they prefer reporting to the attending physician who knows the patient's condition best. Due to the reduced burden of reporting, as it can be brief rather than detailed, there is still a tendency to call the attending physician instead of the on-call physician. For the DHRs to be effectively enforced, collaboration is needed not only within the physicians or clinical department but also with the cooperation of the ward nursing department.	Cooperation with the nursing department is needed.
Q: What are the good aspects and areas for improvement in the DHRs?<br>A: The DHRs have introduced the ON/OFF system due to the 80-hour weekly limit and consecutive work restrictions. Previously, it was autonomously managed, but now, with legal enforcement, schedules are more defined. However, work does not entirely end at 8:00 pm each day; even after leaving, there are still calls from the ward, which can be problematic. Naturally, emergency calls should go to the on-call physician, and it is expected that the attending physician outside the hospital cannot handle them. However, I have heard that ward nurses call the attending physician around midnight to confirm prescriptions for the next day, according to their shift system, even when it is not an emergency. If evening nurses could handle this upon arrival, attending physicians could leave without burden. However, the ward nursing department needs help to change its system. Confirming prescriptions in the evening seems appropriate. Nowadays, attending physicians seem to prescribe earlier, around 4:00 pm, to accommodate this. However, there are still instances where prescriptions are confirmed	Transition period.<br>Cooperation with the nursing department is needed.

around midnight, especially if the ward is busy, and night shift nurses handle it if it has not been completed during the day. It requires much cooperation from the ward.	
Q: How does the nursing department cooperate to operate the ON/OFF system?<br>A: In the XXX department's wards, over 80% of the time, they contact the on-call physician. If they feel the on-call physicians require additional information, they report to the attending physician and receive orders. Nursing department cooperation ensures that orders are issued by the end of the day shift and ward nurses contact attending physicians for confirmation. This process is feasible due to the moderate number of inpatients, ranging from 10 to 18. (Compared to XXX department)	Cooperation with the nursing department is needed.
Q: Nursing departments still contact ward-attending physicians around midnight to confirm prescriptions for the next day rather than adjusting according to the ON/OFF system. Is there room for improvement?<br>A: We have requested the nursing department, not within the XXX department but the ward itself, to have evening shift nurses confirm patient prescriptions quickly. However, they find it practically impossible and respond accordingly. They cite a shortage of nurses and a heavy workload as reasons. In the XXX department's wards, some wards have evening shift nurses who confirm prescriptions, while others rely on night shift nurses. Due to the heavy workload, it is not feasible with the current staffing situation. (Nursing assignments vary by ward.) About 60% of patients in the emergency room are from the XXX department. The proportion of emergency patients is lower than in other departments because these mostly have outpatient-centered admissions. In the XXX department, unpredictability arises because many emergency room patients are admitted, leading to prescriptions being confirmed slowly. Patients are admitted even at 8–10 pm, and to shorten emergency room admission times, patients can be admitted to any ward if beds are available. Other departments might not experience as many emergency admissions, but XXX department's nursing departments feel the workload is heavy, especially during the evening shift. (To address this, would staffing up the nursing departments and increasing the number of XXX department residents be necessary?) The number of XXX department residents is limited. It would be beneficial to increase the number, but realistically, it is challenging. Therefore, most hospitals require and hire hospitalists as dedicated admitting physicians. Additionally, with the reduction in XXX department residents training times from 4 to 3 years, the number of residents has decreased even further. (Would employing hospitalists be helpful?) We have X hospitalists in the XXX and X in the XXX. It is difficult to judge their effectiveness since only a few of X exist. Although having them is better than not, they have relatively light workloads. If the hospitalist system is not activated, it will increase the workload for the supervising faculty, leading to more on-call duties. This would further burden the supervising faculty, possibly	Transition period.<br>Cooperation with the nursing department.<br>Institutional support of the on–off system is needed.<br>Increased demands on faculty roles.<br>Strengthen the workforce of experts.<br>A similar number of patients.

leading them to leave for private clinics and creating a vicious cycle. (It seems the supervising faculty would have insufficient time to focus on residency training. How many patients does each XXX division resident manage?) It is around 25–27 on average. (It seems lower compared to the past. I have seen 40–50 before.) It varies by ward; some have around 35 patients. In the past, they managed patients from both main and extra wards, but now, with ward divisions, they only handle patients from one ward. In the past, XXX division had 35–40 patients, while XXX had around 10, but now, with ward divisions, it is more evenly distributed, averaging around 25 patients. Considering there are more beds now than before, they would manage more patients overall.	
Q: There is concern that limited working hours might decrease the number of patients encountered and surgical cases. What are your thoughts on this?<br>A: Firstly, I do not believe there has been a decrease in patient cases. Even though the number of patients may decrease due to overlaps, the number of individual cases remains similar. (Considering there may be slight variations between patients and surgical outcomes, do you think this level of variation is sufficient within the limited working hours?) Even with variations between patients, as the attending physician, I might not see certain aspects during my on-call shift, but I can address them when I come in the next day. Therefore, I believe it is manageable.	A similar number of patient cases.
Q: How has the actual number of patients you see changed since the enactment of the DHRs?<br>A: (Previously, first-year residents saw more patients than second-year residents). Comparing the workload of first-year residents, it seems that the number of patients they see now is almost similar to what I saw during my first year. I do not think the number of patients for first-year residents has decreased, especially considering the increasing number of ward beds (due to new openings).	A similar number of patient cases.
Q: Previous generations have expressed concern that the patient experience has decreased. They feel there were more opportunities to see patients continuously over 24-hour shifts, but those opportunities have decreased. What are your thoughts on this?<br>A: I do not believe that the DHRs have hindered the education of residents. I do not think resident education is worse than before. Ultimately, the overall number of patients remains almost the same. If one diligently sees patients during their working hours, I do not see the necessity to work late into the night just to see more patients or gain certain experiences. In fact, most treatments are already conducted during daytime hours, and there is ample opportunity for consultation with professors. I doubt that additional night shifts contribute significantly to education. Regarding residency training, learning about medication prescriptions through consultation with professors during the day or discovering additional tests and plans that were previously unknown	A similar number of patients.<br>Daytime patient care and procedures.<br>Focus on daytime work.<br>Discussion with faculty.

seems to be more beneficial for education. I do not believe that the reduction in night shifts has diminished the capabilities of residents from an educational standpoint.	
Q: If you work 12-hour shifts during weekdays, who covers the nighttime shifts on weekdays?<br>A: Weekday 12-hour shifts plus nighttime 12-hour shifts are alternated among the staff, and on Wednesdays, senior staff members take the nighttime shifts. Everyone works daytime shifts and takes on nighttime shifts 2–3 times per week. After a 24-hour shift, they continue working the next day without rest. Currently, even third-year residents in the XXX department take on attending duties. They believe the treatments/procedures and consultations conducted during daytime hours with professors offer more valuable educational experiences. They feel that two nighttime shifts per week are sufficient to gain clinical experience during nighttime hours. Having had sufficient sleep during the night, they approach daytime tasks with a fresh mind, without needing to take naps and take advantage of opportunities to observe, ask questions, and learn from professors during procedures. Being awake reduces the likelihood of errors in patient care, and the knowledge gained during these times stays fresh in their memories.	Senior residents' night shift.<br>Patient care and procedures in the daytime.<br>Focus on daytime work.<br>Discussion with faculty.<br>Less tired.
Q: In the past, resident education involved being on call for 24-hour shifts, where education occurred in a more apprenticeship-style manner, regardless of personal plans. Do you think learning occurs well during breaks?<br>A: With more flexibility (without feeling tense about potential phone calls), residents can explore theoretical aspects or research data organization for cases they want to explore. This flexibility allows for seeing more patients during daytime hours and calmly studying in the evening after work; as for whether such a work system would be beneficial even in a 3-year residency program, in the third year, there is a belief that one needs to study on the go. Presently, due to the limited workforce in hospitals, there is not much time for self-study outside of work hours, so autonomous learning must go hand in hand with work hours. In the past, third-year residents almost functioned at the level of chiefs or fellows, seeing patients and performing procedures extensively. With the reduction in the residency duration to 3 years, the residency program's duties and tasks have been readjusted. First- and second-year residents focus more on inpatient care, while third-year residents mainly focus on outpatient care, MICU, and consulting with other departments.	Search for data.<br>Focus on daytime work.<br>Self-directed learning is needed.<br>Duty system within a team.
Q: In the past, first- and second-year residents used to handle many responsibilities as attending physicians, while third- and fourth-year residents had more time to study and participate in research or perform more specialized clinical tasks. However, now it seems like tasks are distributed equally among residents regardless of their year of training. There is a concern that this might lead to residents not being able to engage	Operation experience in the daytime.

in deeper, more specialized tasks as before. What are your thoughts on this?<br>A: In the XXX department, being a surgical specialty, mastering surgical skills is considered crucial alongside postoperative care. When residents become surgical assistants, the chief resident serves as the first assistant and the attending physician as the second assistant. While postoperative care is important, gaining proficiency in surgical skills is the ultimate goal. Emergency surgeries are often encountered during on-call duties, and since most surgeries occur during the daytime, acquiring surgical skills is prioritized. Therefore, when a resident becomes a fourth-year chief resident, they can serve as a first assistant, ultimately achieving their training goal. Thus, from my perspective, as long as residents can achieve their ultimate training goals, there should not be a significant issue, as the focus is on acquiring surgical skills, which are primarily performed during daytime hours, even within limited work hours.	
Q: Do you plan to remain a fellow for further training?<br>A: Yes. (Would not being a fellow be more challenging?) Actually, fellows and supervisors seem more challenging. However, as a fellow, I am in a position where I need to learn skills, and with many subspecialties, I can rotate through one subspecialty every 6 weeks, allowing me to experience a subspecialty 1–2 times a year. Therefore, if I really want to learn, it is possible during a fellowship. While a fellowship may be challenging, especially at an older age, it is important because mastering skills is crucial for becoming a specialist. Even if it is challenging, it is beneficial if you get to perform many critical surgeries.	A fellowship course is needed to advance the skills.
Q: Do you have plans to remain as a fellow for further training?<br>A: Yes, during the first 1–2 years of training, it was relatively less challenging, so if a fellowship involves difficulties rather than enduring hardships, it can be viewed as an opportunity to hone the skills necessary for personal growth.	A fellowship course is needed to advance the skills.
Q: What do you think is the impact of the DHRs on resident education?<br>A: It is still early to judge whether the DHRs are beneficial or detrimental to excellent resident education, considering that residency training ultimately revolves around education, with both structured learning through textbooks and learning by seeing patients. In the past, residents continuously observed patients while residing in the hospital, which may have provided a different educational experience than the current system, where residents have restricted hours and are divided into ON/OFF shifts with an 80-hour limit. Whether this regulation is helpful or not depends heavily on the capabilities of the individual. It could be beneficial if a resident efficiently addresses every patient's need within the 80-hour limit and then invests the remaining time in personal development. However, if a resident cannot resolve all patient care within the designated hours, it raises doubts about the effectiveness of this regulation. There is likely to be considerable variation depending on individual circumstances. In the past,	Depends on the capabilities of the individual.<br>Self-directed learning is needed.<br>Focus on daytime work.<br>Search for data and research activities in allowed free time.

apprenticeship training exposed residents to patient care duties, intentionally or unintentionally, which might not be the case in the future with the restricted hours. Additionally, in the future it will be crucial for residents to assess what they learned during patient care hours and to plan what else they need to learn. Those who plan well may benefit more. Residents should focus solely on patient care during work hours and utilize the remaining time to learn aspects they have not covered yet. If residents who are unsure of what to do, what they lack, what more they need to study, etc., spend their time without a plan after work hours, it feels like their training time will quickly pass by. While some believe that seeing more patients leads to more learning, others argue that simply seeing more does not necessarily improve the quality of learning. It is more about realizing what needs to be learned qualitatively. Ultimately, it depends on the capabilities of the individual. Supervising physicians may need to assess each resident's shortcomings and assign tasks accordingly. However, considering the workload of supervising physicians, they might need help to fill the gap left by residents, leading to increased educational burdens.	
Q: What do you think about the residency training after the DHRs?<br>A: Previously, I juggled patient care with preparing for presentations or studying for the next day, and sometimes I could not get enough sleep. Currently, even if I leave work late on my days off, I have free time (without criticism, guilt, or interruption) to hand over calls to the on-call physician and work at my own pace. Although it still feels overwhelming to handle third-year tasks during the transition period (such as my own department tasks as a chief resident, ICU care, and covering the ER shift for the second-year resident), I anticipate this to improve as first-year residents become third-year residents in the future.	Transition period.<br>No worries, complete freedom from work.<br>Preparation of next work.<br>The task was transferred to senior residents.
Q: What is residency like after the DHRs?<br>A: Previously, I used to oversee up to 30 NICU patients. In Seoul, staff numbers are already high, so each resident typically oversees 10–15 patients. While I used to intend to look up patient-related queries, often, due to time constraints, I could not research them thoroughly and had to move on. With fewer inpatients/assigned patients now, there seems to be more time available to investigate patient inquiries, which is beneficial. Concerns appeared when transitioning from the second year, where I only worked in the ER, to the third year, where I had to start working in the ICU. I worried whether I could supervise the tasks of second-year residents, as in the past, second-year residents already had experience in both the ER and ICU, so by the time they reached their third year, they could supervise first and second-year tasks. However, now, having to tackle tasks in the ICU without prior experience made me question whether I could also back up the ER. (Q: Would it not have been more burdensome for second-year residents to handle both ER and ICU simultaneously?) Second-year	Search for data and research activities in allowed free time.<br>Gradual acquisition.

residents could receive backup support from third- and fourth-year residents. In practice, there were not any major issues. I believe tasks were gradually escalated, allowing ample time for adaptation, albeit slightly delayed.	
Q: Do you believe residency training is adequately conducted?<br>A: Yes, learning happens amidst the hustle. Since our patients are mostly XXX, who are generally sensitive, both residents and chief physicians tend to be vigilant, resulting in a learning environment even during reprimands. It seems like we learn more through mistakes and trial and error. Learning feels tangible, similar to learning by doing. (There are concerns that with the reduction in training time due to the DHRs, there might be fewer opportunities for clinical experience and training. What are your thoughts?) In our department, while the ON/OFF schedule is fixed, residents from the first to fourth year rotate through the wards, ER, and ICU during the daytime, so I believe it is adequate. Sometimes, if residents do not get enough sleep during their night shifts, they are allowed to take a nap for about three hours during the daytime. Residents from all levels work side by side during the daytime, making it easy to ask each other questions. (Is the third year not almost like being an attending physician during the daytime?) Third-year residents work in a more independent space, waiting until issues arise and then stepping in to assist or taking on the role of chief when the chief physician participates in surgeries. While third-year residents still have plenty of break time, they now have more responsibilities than before. They have more duties, such as providing backup in outpatient clinics or rotating through ward duties. Third-year residents in the XXX have duty shifts four times a week on Mondays, Wednesdays, Fridays, and Saturdays, while in the XXX, they have duty shifts twice a week. In the XXX, there are two third-year residents, so they share duty shifts on Wednesdays and alternate for the remaining three days, meaning they each have duty shifts three times a week. Originally, duty shifts were shared among third- and fourth-year residents. In the XXX, which is a specialized department, third- and fourth-year residents work together to cover the wards. There are always at least three residents on duty in the XXX, even during vacation seasons, sometimes two when two residents are on leave. Duty shifts are evenly distributed among different year levels, such as 1–2–4, 1–3–4, 2–3–4, etc. It would not run smoothly without this kind of schedule, especially during the night shifts.	Not alone on the night shift.<br>Duty system within a team.<br>Patient care and procedures in the daytime.<br>Discussion of patient care.<br>The task was transferred to senior residents.<br>Rotation of night shift within a team.
Q: In the ON/OFF system, from the perspective of a resident, it seems that reporting takes precedence over decision-making. What are your thoughts on the educational aspect of decision-making for residents?<br>A: Residents are not making decisions independently. If a resident intends to engage in decision-making, I am willing to support them, but I do not consider it an obligation. The final decision should not be made solely by the resident but rather after reporting	Unchanged faculty role of final decision.<br>A fellowship course is needed to advance the skills.

to and consulting with the supervising specialist. Since residents rotate through different duties every 12 hours, there needs to be more continuity. For example, a patient seen in the ICU yesterday might be seen by a different resident today, which limits the ability to make significant decisions. Therefore, the supervising specialist should be the one making these decisions. (From the perspective of a resident, while they may understand the general flow, they might not be aware of all the details. Do you believe resident education is adequately conducted?) Compared to the past, it might be considered lacking. Therefore, there might be a need to supplement residency training through fellowships. I also anticipate that there might be changes in the residency training process in departments such as the XXX. Perhaps they will adopt a system similar to the one in the United States, where residents complete a three-year program followed by a two-year fellowship.	
Q: How do you perceive resident education since implementing the DHRs?<br>A: Patient exposure has decreased. With reduced training hours and increased sensitivity to patient safety and medical errors, the supervising faculty is less likely to delegate critical tasks to residents, such as caring for severely ill patients or performing complex procedures. Decision-making opportunities have decreased, especially for first- and second-year residents. Tasks that were previously performed by junior residents are now often delegated to senior residents. For example, I had much experience with XXX as a first-year resident. However, now first-year residents typically do XXX in the latter part of their first year or under the supervision of senior residents and supervising faculty. Even tasks such as XXX, which residents performed once, are now typically conducted by fellows or higher-level trainees. Overall, there has been a decrease in what residents can learn over the four years compared to ten years ago. Both patient and procedural experiences have declined and do not meet the standards for adequate education. In departments such as XXX, learning occurs through patient experiences. However, with reduced patient exposure, residents may not develop the skills necessary for higher-level decision-making positions in the future. Regarding curriculum development for each year, most of the content outlined in the training objectives provided by professional societies is satisfactory. However, residents may not have the opportunity to engage in tasks of higher difficulty levels that they could have performed in the past. In summary, while basic competencies are met, more advanced, specialized skills may be lacking.	Decreased patient experience.<br>A fellowship course is needed to advance the skills.<br>Can acquire basic core competencies.
Q: Compared to the past, it seems that both patient exposure and the capacity to perform various treatments, procedures, and surgeries have decreased in the residency training process. Should we view this as a reduction in the quantity of training or a decline in the quality of training?<br>A: It is true that the quantity of training has decreased, and it seems that the quality has	Decreased resident education.<br>Can acquire basic core competencies.<br>Self-directed learning

also declined. From the perspective of residents, they may perceive an improvement in training conditions compared to the past, as they now have more time for study. However, from the perspective of supervising specialists, when comparing the ability of residents to manage similar patients, it is evident that it has declined compared to the past. This includes aspects such as patient assessment and developing future treatment plans. In the past, if residents were struggling to keep up, the supervising specialist would provide more intensive training, guidance, or explanations. However, supervising specialists are now less likely to invest the same effort.	is needed.
Q: How do you perceive residency training since implementing the DHRs?<br>A: In the past, there was more comprehensive education covering clinical practice and research, but now the focus is mainly on patient care. However, few individuals complete a fellowship after residency. Moreover, with the reduction from 4 to 3 years, the emphasis is on acquiring basic skills as a physician in the XXX field. In my opinion, the quality of residency training has declined. Regarding concerns about the potential impact on patient care decision-making skills upon transitioning to private practice, it is difficult to make a definitive judgment. Previously, residents typically gained sufficient experience during their training in university hospitals and were adequately prepared for private practice. However, there might be a gap in the accumulation of relevant experiences for private practice. In the XXX specialty, where there is ample training, there can be significant discrepancies in competency between exceptional and less proficient residents. Nevertheless, assessing overall patient care delays without further objective data is challenging. Previously, efforts were made to train residents regardless of their capabilities, although there now seems to be a reduction in such efforts and time investment in supervision beyond the minimum requirements. The level of supervision may vary depending on the capabilities of the individual.	Can acquire basic core competencies.<br>Depends on the capabilities of the individual.<br>Self-directed learning is needed.
Q: How do you perceive residency training since implementing the DHRs?<br>A: After the DHRs were implemented, attending physicians responsible for hospital wards received calls for all wards. The on-call team handles the surgical duties. Ward duties are assigned to one person, and outside regular hours, the on-call team (consisting of three members) handles emergency room, surgery, and ICU care. In the XXX specialty, ICU calls are directly received by the supervising specialists. There are no attending physicians stationed in the ICU. The number of ICU patients per month in each department ranges from 1 to 2. In most cases, the ICU is supervised by anesthesiologists, so it is more about taking calls and attending rather than continuously overseeing while on duty. First-year residents typically oversee the wards, and second-year residents oversee the emergency room. They do not stay on-site outside regular hours. In the XXX and XXX specialties, although the ICU is crucial, XXX is more important, so if there is a problem in the surgical ICU (SICU),	Rotation of night shift within a team.<br>Increased demands on the faculty roles.<br>Gradual acquisition.

they contact the attending physicians. They also contact the supervising specialists during regular hours, not just outside regular hours. Attending physicians might be in surgery performing surgeries, meaning they directly contact a supervising specialist. Therefore, we feel that the workload is gradually increasing compared to the past. As a result, their preparation process for becoming specialists needs to catch up compared to before. Compared with the past, residents at the same level might have slightly less experience. However, it is not certain that the quality has decreased. It does not seem inherently deficient. They are proficient in performing procedures.	
Q: What do you think about the idea that residents can utilize their off-hours to study more due to the time restrictions?<br>A: I am still trying to figure that out. From my perspective, I saw two potential benefits of the residency time restrictions. First, it ensures enough rest for residents, and second, it was thought that it would allow residents to engage in more self-directed learning and research activities. However, in reality, residents seem to spend more time on leisure hours. Since autonomous time is provided outside regular hours, it varies depending on the capabilities of the individual.	Improved life.<br>Depends on the capabilities of the individual.<br>Self-directed learning is needed.
Q: With improvements in the residency training environment, third-year residents are now solely responsible for managing the ICU and delivery room. Do supervising specialists also feel a greater sense of stability?<br>A: In the XXX, half of the ICU is managed by second-year residents, while third-year residents manage the other half. For example, in the CCU, the second-year residents handle it (connected to the ER, although on-call is separate), and in the MICU, it is solely managed by third-year residents. (Do you think the transition from second-year to third-year senior roles is delayed?) The timing has indeed been pushed back compared to before. In the past, first-year residents handled the ward, ER, and ICU. The preparatory process is progressing more slowly than before.	Gradual acquisition.
Q: How are third-year residents performing now that they have to make decisions about patients? Should they be quite skilled by this point?<br>A: I believe that third-year residents are capable. In the XXX, if a resident could make decisions about patients by the early part of their second year, they were trusted to do so. By now, as third-year residents, they are proficient. Ultimately, they achieve their intended position when they reach their third year. However, residency training for specialists is less in-depth than it used to be, only reaching the minimum competency level. They may not reach the same depth of skill as before. Normally, the more experienced individuals tend to perform better since they learn more from their mistakes.	Gradual acquisition.<br>Can acquire basic core competencies.
Q: There was also dissatisfaction with the fact that surgical experiences could not be fully observed because the restricted working hours did not provide much benefit, as	Simply restricting training hours does

it is important to observe surgeries through to completion, especially when they require long durations. What are your thoughts on this matter?<br>A: Understanding the intricacies of XXX surgery is challenging, even for fellows. Witnessing a few XXX surgeries to completion does not necessarily mean complete comprehension. While one can grasp the concepts from books, experiencing certain aspects of the surgery once or twice does not equate to full understanding. XXX surgery involves the integration of various surgical techniques. It requires an understanding of the anatomical structures, changes in structures due to complications, determination of surgical indications, and knowledge of anatomical variations. Experience is essential in mastering these aspects. XXX surgery, being a highly specialized field, is not easily mastered even during the entire residency training process.	not necessarily imply a setback in resident education.
Q: What do you think about the impact of the DHRs on residency training and clinical experience?<br>A: Even simple surgeries are being performed by fellows and clinical instructors, and in the XXX, fellows and clinical instructors are constantly present, which means there are fewer opportunities for residents. It is rare for residents to assist in surgeries from start to finish. First-year residents, both in the past and now, have very few opportunities. The restricted training hours have limited exposure to surgeries; however, it does not feel unfair. The role of residents remains similar, and there is little difference from the previous residency programs. It is inaccurate to say we have learned less due to the 80-hour restriction. Basic medical procedures and surgeries for primary care are adequately learned during residency, and the more complex procedures are typically acquired through fellowships or under the supervision of specialists.	Can acquire basic core competencies.<br>A fellowship course is needed to advance the skills.
Q: Has the curriculum for residents in their first to fourth years changed due to the clearer implementation of the ON/OFF system?<br>A: The overall framework has mostly stayed the same. I believe residency education remains similar to before following the implementation of the DHRs. (How long has the ON/OFF system been in place?) I heard that the ON/OFF system was established in the XXX before the DHRs were implemented, although I am not entirely sure. (Was there definitely an OFF system when you were training in the past?) There was no official OFF system; it was more of a standby status for 24 hours. When a resident was part of the on-call team, they worked; otherwise, they rested as needed. We were always on standby. The ON/OFF system was implemented experimentally in anticipation of the 80-hour workweek. (Are there annual training goals set either by the hospital itself or by professional societies?) I understand the training content is documented annually on the respective specialty board's website and sent	Unchanged residency training curriculum.<br>Can acquire basic core competencies.

electronically. Following this, eligibility for the specialty board exam is granted.	
Q: Eventually, in the case of the XXX specialty, tasks are evenly distributed from the first to the fourth year, so now it seems that both the 3-year residency program and the 2-year fellowship program must be completed to feel capable as a specialist. What about XXX's specialty?<br>A: Surgical techniques are becoming minimally invasive, and both patients and practitioners are leaning towards it. Specialized skills can be learned during fellowship training. So, I agree with this statement to some extent. However, traditional surgical methods (such as laparotomy) remain part of the residency training program, and one can continue to perform them after completion. Even though laparoscopic procedures are replacing major surgeries, there is still a need for abdominal surgery, which is why fellowship training is necessary. In the past, appendicitis was often treated with laparotomy, making it manageable within the residency program. However, following the present shift to laparoscopy, mastering such techniques requires fellowship training.	Can acquire basic core competencies.<br>A fellowship course is needed to advance the skills.
Q: Has the redistribution of tasks been implemented, and would the redistributed tasks, especially on-call duties, not burden the upper-year residents?<br>A: Since patient care in the ward has been managed collectively by the team, consisting of residents from the first to fourth years, or sometimes from the second to fourth years, even if tasks are handed over, the upper-year residents who take over the on-call duties are already familiar with the patients, so it does not pose a significant burden.	The task was transferred to senior residents.<br>No burdensome work.
Q: Does it seem like there is no burden with upper-year residents taking on-call duties compared to before?<br>A: Even if upper-year residents take on-call duties in a different rotation, they have already received training previously, and as the patient's progress (post-surgery management) is similar, they only need to handle unusual (risky) cases well. (What about the quality aspect of patient care?) Professors have always received patient reports and made decisions on emergency surgeries, meaning the reporting may change, although the final decision-making remains with the professors. Previously, the attending physician reported, yet now it is the on-call resident, while the decision-making still lies with the professors, so there does not seem to be any change in this aspect.	No burdensome work.<br>Unchanged faculty role of final decision.<br>Only change of reporter.<br>Increased awareness of patient safety.
Q: How is patient handover performed at the end of the shift?<br>A: The on-call resident hands over the patients to the ward duty resident before leaving. Ward duty responsibilities rotate among residents from years 1 to 4, with each resident typically serving duty around twice a week. Even the fourth-year residents have similar duty schedules. There is no concept that lower-year residents see more patients. With the department divided into X sections, each year group, such as first-year and second-	Senior residents' night shift.<br>Rotation of night shift within a team.

year residents, is responsible for their designated section. During the day, the ward duty resident receives primary calls from the ward and the emergency room (ER); meanwhile, the fourth-year residents mostly work in the operating room and rarely take primary calls but still receive the reports.	
Q: Are there any significant challenges in handing over patients, considering that the attending physician previously had a complete understanding of the patients and now needs to hand them over to the on-call resident?<br>A: In the XXX, as the clinical course of patients (surgery, postoperative care, recovery, etc.) tends to be similar, there are no significant challenges. If there are particularly critical patients, plans are communicated in advance via phone or provided in the progress notes as needed. (Q: Have there been instances where patient conditions worsened due to the handover process?) Such instances are rare. The professors also receive patient condition updates, meaning they are adequately informed to supervise. Moreover, immediate communication and response ensure that patient care is not significantly compromised.	Increased awareness of patient safety.<br>Unchanged faculty role of final decision.
Q: How do you think patient safety is affected by the handover process in the ON/OFF system?<br>A: If the handover process is carried out effectively, then I do not think there would be any issues. At the early stage, when the settlement had not been properly established, there were times when even senior residents had doubts, such as "Would a junior resident delegate tasks to me?' Due to long-standing customs, there have been instances where senior physicians were not physically present in the hospital, assuming that junior residents would handle patient handovers. It was a bewildering experience to realize that the senior physicians were not in the hospital before returning to find them there. However, as awareness that not being present in the hospital during duty can lead to significant problems grows, I anticipate no issues as long as the on-duty resident is present and attentive according to the ON/OFF system. As the residents progress through their training, this ON/OFF system becomes more established, and handovers and duty rotations are likely to occur more smoothly, posing no significant concerns for patient safety.	Transition period.<br>Increased awareness of patient safety.<br>A clear handover is needed.
Q: Would it not be risky if a patient's condition deteriorates during the handover?<br>A: The ON/OFF system is still not firmly established, but if a patient's condition worsens during handover, the assigned resident may continue to care for the patient even late into the night. For example, this might happen with patients in the intensive care unit or those undergoing ECMO treatment. Occasionally, the resident may accompany the patient in the ICU while handing over care. However, since they need rest for the next day's shift, they eventually complete the handover and leave for home. While most shifts end by 6 pm, the handover process often starts at that time, so leaving	Rotation of night shift within a team.<br>Patient safety is a priority.

strictly at the end of the shift is not always the case. Therefore, it is not necessarily considered risky for a patient during the handover time.	
Q: There have been concerns about whether patients might be adversely affected during the ON/OFF handover time, especially if the handover is not conducted properly (as it is not easy to grasp the full picture of the patient's condition). How do you view this issue?<br>A: In the XXX department, teams are assigned based on seniority levels. There are no instances where the XXX team is omitted among the on-call physicians, and efforts are made to ensure that team members do not overlap, with a focus on assigning physicians who are well-versed in the conditions of the ward, especially in the XXX. The handover sequence prioritizes patients with unstable conditions. Unless there is a new emergency admission, the chiefs are already familiar with the patients admitted to the unit. We take pride in our department's familiarity with our inpatients and believe patient safety is not compromised during handovers, especially in the XXX, where residents from first to fourth year work together during the daytime and are already familiar with the patients, regardless of who is on duty. There is always ample backup support from experienced fourth-year residents, so issues are unlikely to arise. (What about the roles of supervising specialists and professors?) When fourth-year residents find a situation challenging, they usually report it to the professors, proceed with medication, and if they feel something is wrong, the professors intervene immediately. (How about other divisions?) XXX also operates in teams, rotating through ward patient rounds, so they are well-versed in all patient situations. Even if a first-year resident from XXX is off-duty, there are still second or third-year residents available to take over ward calls. The XXX department is divided into XXX, XXX, and XXX units, operating as separate teams. Each resident is assigned to a specific unit with only X residents.	Rotation of night shift within a team.<br>Patient safety is a priority.<br>Efforts to prevent gaps in patient care.<br>The faculty role has the final decision.
Q: What do you think about the impact of the DHRs on patient safety?<br>A: There are two contrasting perspectives to consider. Firstly, it is believed that the attending physician, who works during the day, should continue the overall plan for the patient in consultation with the professors. However, it is seen as challenging to fully grasp what happened outside of one's duty hours solely through handover notes regarding changes in the patient's condition. There is concern about whether it is possible to understand the patient fully within the mandated 80-hour workweek. Secondly, there are instances where continuous exposure to a patient may lead to overlooking changes, but taking a break and returning might make these changes more apparent. In terms of patient safety, although there may be limitations in fully grasping the patient's condition, taking breaks could lead to noticing new changes in the patient's condition upon returning. It is thought that having proper rest would result in fewer	Concerns about discontinuity for patient care.<br>Residents' well-being is related to patient safety.<br>Less tired, fewer mistakes.<br>Focus on daytime work.

errors. If a resident's condition is not optimal, it could pose a greater threat to patient safety. Therefore, while there may be drawbacks to not fully understanding the patient, there could also be advantages in noticing new changes upon returning after a break and gaining insights from the perspective of the person handing over the patient	
Q: Some express concerns about the interruption in the continuity of care by residents returning after a break) and the duty hour system, which necessitates handovers. They wonder if resident education and patient safety can be adequately maintained. What are your thoughts on this?<br>A: Many share such concerns. There are differing views on the interruption of the continuity of patient care and the inadequacy of resident education hours. Some individuals hold firm opinions on these matters, which may not align with others. Regarding patient safety, there is now a greater emphasis on faculty members being attentive. As long as faculty members have a thorough understanding of the patients' conditions and residents fulfill their duties of patient care and reporting during their working hours, it is believed that patient safety will not be significantly compromised. However, suppose supervisors are seen to be monitoring effectively. In that case, there may be pushback, with questions about whether supervisors should continue to provide care directly or be expected to work without breaks. The role and burden of supervising physicians need to increase. Ultimately, it is felt that the number of supervising physicians should increase proportionally to the number of residents. If the working hours of residents have been reduced, then the workforce should be expanded accordingly. Current solutions include increasing the number of physician assistants (PAs) or hospitalists, but ultimately, it is believed that more specialist physicians need to be recruited. When it comes to hiring specialists, some have been appointed at a level below clinical instructors, but realistically, there are limitations to how far this can go. Regarding the dedicated attending physician system, it is deemed necessary to increase the number of specialists who can take responsibility for patients, both among supervising physicians and residents. However, this expansion needs to occur nationally rather than solely within individual hospitals. If hospitals cannot recruit more faculty due to financial constraints, the increased faculty workload could negatively impact patient safety. To enhance patient safety, the working hours of residents should remain restricted, but the number of specialist physicians should increase. In advanced countries such as Australia or those in Europe, resident working hours are limited to 35 hours per week for all workers, not just residents. Therefore, it is believed that similar working hour restrictions should apply to specialist physicians as well. If the government prioritizes patient safety, it should impose working hour restrictions on specialist physicians and bolster their numbers. In the current structure, if some individuals reduce their workload, others end up working more. Thus, a	Increased demands on the faculty roles.<br>Strengthen the workforce of experts.<br>Increased awareness of patient safety.

situation ensuring that the working hours of all healthcare providers involved in patient care are guaranteed would ultimately benefit patient safety in the long run.	
Q: Many had concerns about the DHRs, but what impact do you think it has had on actual patient care?<br>A: The DHRs were implemented immediately, but in reality, preparations have been gradually underway for two years. Despite initial concerns, things seem to be going well. Professors were worried about the continuity of care, especially in the ER and ICU, where there needed to be at least one resident on duty per division. However, these concerns have yet to materialize significantly. Instead, residents seem more diligent because of their duty obligations, which is positive. Initially, when the regulation was first enforced, there were instances where residents on ICU duty left the hospital because no patients required immediate attention. However, this issue has improved after attention was drawn to it. Previously, residents sometimes took free time following a quick resolution to their work, and patients were stable. However, they now feel it is better to be responsible and work during duty hours since they are no longer criticized during their off-duty hours. They evaluate themselves as better able to handle the responsibility when on duty. In the ICU, where it was always difficult to assign one person to cover duty within the 80-hour workweek limit, it is now being performed correctly. While it was challenging to schedule, it is now being adhered to. Overall, it is believed that everyone is happier with the current system. In departments without critical care patients, even third-year residents have to cover duty every three days, which some see as a disadvantage. Some argue that if the 80-hour workweek limit is unmet, one person should be removed from the ICU duty roster. However, overall, the XXX department feels more secure. Instead of being called in from elsewhere in the hospital, residents now come directly to the ICU duty room, which is much better for patient safety. In the ICU and hematology departments, residents rotate duty among first to fourth-year residents. In other departments, duty is assigned by rotation and experience level across the wards, ER, and ICU. This means duty residents cover all departments, leading to better patient care as residents no longer become fatigued and fail to assess patients properly. There are no longer cases of residents leaving unnoticed. With duty shifts lasting 24–72 hours, one resident is always responsible for patient communication, eliminating situations where patients were not properly attended to because no one received their reports.	Less tired, fewer mistakes.<br>Efforts to prevent gaps in patient care.<br>Increased awareness of patient safety.<br>Rotation of night shift within a team.<br>Not alone during a night shift (all training years residents on duty).
Q: Has there been any significant difference in patient care before and after implementing the ON/OFF duty system and the DHRs?<br>A: There has been no significant difference. However, there have been concerns within the XXX department. Initially, there were often four residents on duty together in March, April, and early May, comprising first to fourth-year residents. This was	Rotation of night shift within a team.<br>Efforts to prevent gaps in patient care.<br>The task was

because the first-year residents were not yet proficient in their roles and had limited procedural skills. However, now that only three residents are on duty, there is some anxiety. The reduction in the number of residents participating in duty across the first, second, and fourth years has contributed to this. Until August, all fourth-year residents participated in duty. From September onwards, as fourth-year residents left, there was a decrease in the workforce. Previously, senior residents (third and fourth years) would wait for on-call duty, but this was rare. The training hour limit did not restrict them, and they typically worked 60–70 hours a week. If they were called in, they were competent enough to handle consultations, perform ultrasounds, and report findings independently. However, the sense of security that came with being on-call has disappeared since the DHRs. Although not common, having designated on-call personnel provided reassurance that patients could be attended to in emergencies. Regarding the workload, while the workload of first-year residents has decreased, it has increased for third- and fourth-year residents. However, the increased workload is perceived as reasonable rather than burdensome and is generally accepted. Regarding the department's atmosphere, it seems to align with the current trend among residents to support each other, even if it means taking on more tasks. Although having increased responsibilities may not be ideal, residents seem to be understanding and accepting. In the XXX department, third and fourth-year residents have been participating in duty rotations roughly two to three times a week, even before the DHRs. Therefore, there has been no significant difference following the law's implementation. The DHRs and restrictions on the maximum number of duty hours and rotations have reduced the number of duty assignments for first-year residents but remained similar for third and fourth-year residents. On-call duty used to involve third and fourth-year residents supporting first and second-year residents, but now it mainly refers to hospital-based duty rotations.	transferred to senior residents.
Q: In the long run, if the quality of residency training declines, how do you think it will affect patient safety?<br>A: If the current residents do not undergo fellowship training before leaving their residency, it could negatively impact them because they would lack sufficient experience. Most surgical procedures are typically performed by fellows, so if residents do not gain that experience before leaving, their competency would undoubtedly be lower. When practicing in private hospitals, it is essential to discern which patients require your attention and the cases you can handle. Similarly, the ability to assess the feasibility of surgeries is crucial. Without completing a fellowship, I believe the quality of patient care could suffer. Ultimately, this could have a detrimental effect on patient outcomes. During my first year of residency, I experienced working in a busy ward where I barely had time to sleep for an hour a day due to the	A fellowship course is needed to advance the skills.<br>Increased awareness of patient safety.

workload of managing 30–40 patients. This lifestyle did not feel right to me, and I believe it was also detrimental to patient safety. While the current restrictions on training hours may not directly impact patient safety during residency, when residents become specialists and transition to private practice, their competence may not meet the required standards, resulting in inadequate patient care. Therefore, if residents do not receive comprehensive training, it could compromise patient safety in the long run.	
Q: Do you think the 80-hour workweek limit benefits patient safety?<br>A: There is a concern that residents may become too fatigued from the intensity of their work, which could lead to errors in patient care and treatment decisions. Patient safety is closely related to the quality of the care provided, and it is evident that the quality of the care being delivered by current residents may not match that of previous years. While major medical errors may not be prevalent, deficiencies in knowledge and experience could impact patient care in smaller, nuanced ways. Both inadequate knowledge and lack of patient experience could contribute to this situation. Due to the 80-hour workweek limit constraints, residents are gradually transitioning from specialty-based to ward-based work. This shift, combined with a higher patient volume than the number of residents, means that adequate education and training may not be fully achieved in the ward-based setting. As for potential solutions, there have been efforts to improve the training curriculum within the department. For example, the XXX society has developed core competency guidelines for XXX, including patient volume targets, essential clinical symptoms, signs, procedures, and a list of conditions with which residents should be familiar. Evaluation methods have also been updated to include slide assessments, written exams, and oral and bedside performance evaluations. Assessments are now conducted within one month of the end of each year of training, and there is a move towards a pass/fail grading system. Additionally, there is a plan to shift away from traditional board exams towards workplace assessments to ensure competency. Despite these efforts, some still feel that the competency of residents may not match that of previous years. However, it is worth considering that residents today have access to numerous study materials and resources, such as textbooks, online resources, and conferences, which may compensate for decreased patient experience. Overall, while there may be concerns about the impact of the 80-hour workweek limit on patient safety, ongoing improvements in training methods and the availability of resources offer hope for maintaining high standards of care.	Residents' well-being is related to patient safety.<br>Less tired, fewer mistakes.<br>Can acquire basic core competencies.<br>Supervisor's role in resident education.<br>Self-directed learning is needed.<br>Search for data and research activities in allowed free time.<br>Gradual acquisition.
Q: Many say that the lives of residents have improved significantly, and while the quality of residency training might not be as robust as before, foundational education is still being provided. What are your thoughts on the quality of patient care and patient safety?<br>A: In our department, patient safety has actually improved because faculty members,	Faculty role in the final decision.<br>Increased demands on the faculty roles.<br>Can acquire basic

rather than residents, directly handle patient care. Although the workload for supervising attendings has increased, decisions regarding patient care are made promptly, which I believe has enhanced patient safety. As for residents transitioning to outpatient settings for patient care after completing their training, I think they generally handle primary care responsibilities adequately, considering the potential challenges that may arise in outpatient or primary care settings. Regarding the difference between university hospitals, where supervising physicians oversee patient care decisions directly, especially in critical care units such as the ICU, and the scenario where residents transition to outpatient settings after training, concerns have been raised about the residents' abilities to assess patient conditions and severity accurately. (Some fear that delays or errors in patient care might occur. What are your thoughts on this?) Personally, I do not share those concerns. I believe that completing residency training in my specialty equips residents with the ability to assess patient conditions competently. Regardless of the 80-hour workweek restriction, I think residents develop the necessary skills to judge patient statuses effectively. Without such training, there could be inherent risks and criticisms, potentially leading to setbacks in outpatient practice. Therefore, I do not anticipate significant differences in patient safety outcomes.	core competencies.
Q: Are there any positives or areas for improvement regarding the DHRs?<br>A: Before the regulation was enforced, there was a small amount of unease about leaving work. For instance, there were concerns about whether to stay in the hospital if an ICU patient's condition was unstable. However, after the regulation came into effect, such situations are now guaranteed by regulations, and even professors inquire about why someone is not leaving, which seems like a positive change.	Guaranteed OFF time.<br>No worries.
Q: Is there anything else you would like to add regarding the positives or areas for improvement after the DHRs?<br>A: Due to our division's limited capacity, possessing only two resident positions, it was challenging for two residents to adhere to the 80-hour limit. However, with the arrival of a fellow, they now take turns serving duty shifts, with two residents covering shifts from Monday to Saturday and Tuesday to Sunday, which has improved the situation. The fellow also takes primary calls in the emergency room. In fact, when XXX assessed the residents' working conditions last year, they honestly reported working 84 hours, which led to intense monitoring. Since then, there has been a significant improvement, and efforts have been made to adjust the residents' working hours to 78–80 hours or less. There was a time when our department faced a crisis due to a significant reduction in resident support, which prompted us to redistribute responsibilities effectively. Therefore, it seems that the implementation of the DHRs has been carried out smoothly. Our department's support decreased this year, and one	Supervisor’s night shift.<br>Constant efforts for resident life and education.

person resigned, prompting further restructuring. Despite adhering to the 80-hour work limit and maintaining duty days, the restructuring caused a slightly increased workload.	
Q: Do you have any further comments regarding the positives or areas for improvement concerning the DHRs?<br>A: Improvements should have been made sooner in terms of residents' lives. Priority should have been given to improving residents' lives, and residents should receive enough rest. Additionally, improvements are still needed in the lives of specialist physicians. (Do you think hiring PAs or hospitalists is necessary for staffing reinforcements policy-wise?) Most likely both. What is immediately noticeable is the hiring of PAs. Some hospitals have implemented dedicated admitting physicians, which I have experienced, which were inefficient. When their regular hours ended, they would leave immediately after their shift. They hold the status of specialist physicians, meaning the next person they need to hand over to is a resident. There could be gaps in handovers, or errors could occur during the transfer process. There is also a subtle discomfort in feeling like I should also be doing this as an attending physician or specialist. No clear guidelines define roles, so sometimes they work for one or two months and then leave. From the perspective of XXX, PAs or surgical assistants feel better. Specialists want to be hired in positions where they can leverage their expertise. That is, they want to be hired in positions where the scope of their role includes caring for ward patients and performing surgeries if they are surgeons. It would benefit the hospital to have mutual assistance between supervising physicians and specialists. Ultimately, there is a need for staff reinforcement of specialists within the hospital.	Increased demands on the faculty roles.<br>Strengthen the workforce of experts.
